# Efficient Removal of Tetracycline from Water by One-Step Pyrolytic Porous Biochar Derived from Antibiotic Fermentation Residue

**DOI:** 10.3390/nano14171377

**Published:** 2024-08-23

**Authors:** Xinyu Zhao, Guokai Zhu, Jiangtao Liu, Jieni Wang, Shuqin Zhang, Chenlin Wei, Leichang Cao, Shuguang Zhao, Shicheng Zhang

**Affiliations:** 1 Miami College, Henan University, Kaifeng 475004, China; 2138030018@henu.edu.cn (X.Z.); zgk0317@henu.edu.cn (G.Z.); ljt4015@henu.edu.cn (J.L.); jieniwang@henu.edu.cn (J.W.); zhangshuqin@henu.edu.cn (S.Z.); wcl2000@henu.edu.cn (C.W.); 2 College of Chemistry and Molecular Sciences, Henan University, Kaifeng 475004, China; 3 Huaxia Besince Environmental Technology Co., Ltd., Zhengzhou 450018, China; zhaoshuguang@besince.cn; 4 Shanghai Key Laboratory of Atmospheric Particle Pollution and Prevention (LAP3), Department of Environmental Science and Engineering, Fudan University, Shanghai 200433, China; zhangsc@fudan.edu.cn

**Keywords:** antibiotic fermentation residue, biochar, tetracycline, adsorption

## Abstract

The disposal and treatment of antibiotic residues is a recognized challenge due to the huge production, high moisture content, high processing costs, and residual antibiotics, which caused environmental pollution. Antibiotic residues contained valuable components and could be recycled. Using a one-step controllable pyrolysis technique in a tubular furnace, biochar (OSOBs) was produced without the preliminary carbonization step, which was innovative and time- and cost-saving compared to traditional methods. The main aim of this study was to explore the adsorption and removal efficiency of tetracycline (TC) in water using porous biochar prepared from oxytetracycline fermentation residues in one step. A series of characterizations were conducted on the prepared biochar materials, and the effects of biochar dosage, initial tetracycline concentration, reaction time, and reaction temperature on the adsorption capacity were studied. The experimental results showed that at 298 K, the maximum adsorption capacity of OSOB-3-700 calculated by the Langmuir model reached 1096.871 mg/g. The adsorption kinetics fitting results indicated that the adsorption of tetracycline on biochar was more consistent with the pseudo-second-order kinetic model, which was a chemical adsorption. The adsorption isotherm fitting results showed that the Langmuir model better described the adsorption process of tetracycline on biochar, indicating that tetracycline was adsorbed in a monolayer on specific homogeneous active sites through chemical adsorption, consistent with the kinetic conclusions. The adsorption process occurred on the surface of the biochar containing rich active sites, and the chemical actions such as electron exchange promoted the adsorption process.

## 1. Introduction

With the continuous development of modern medical technology and the improvement of living standards, people are paying more attention to their health. Antibiotics, as one of the most commonly used drugs worldwide, play an essential role in medical treatment due to their special bacteriostatic and bactericidal effects and are widely produced and used. From 2000 to 2015, global antibiotic consumption rapidly increased from 21 billion doses per day to about 35 billion doses, a growth of 65%. China is the major user and producer of antibiotics [1]. It is estimated that China is the world’s largest antibiotic product market, consuming more than 25,000 tons of antibiotics annually [2]. Tetracycline (TC) is widely used due to its activity against most Gram-positive and Gram-negative bacterial strains, protozoan parasites, as well as atypical organisms such as mycoplasmas, chlamydiae, and rickettsiae, and its relatively low price [3]. The use of tetracycline antibiotics has significantly improved public health and promoted the development of the livestock industry but has also brought serious issues with antibiotic wastewater. The widespread use of antibiotics has led to their widespread presence in the environment, and raised concerns about ecological security. At the same time, the aging microplastics in the environment have become a potential pollutant carrier. These microplastics could not only adsorb a variety of pollutants including antibiotics, but also had the ability of long-distance transportation in the aquatic environment, which increased the risk of pollutant diffusion and accumulation, and posed a serious threat to the health of the aquatic ecosystem [4].

Currently, antibiotic residues are widely present in wastewater and enter the aquatic environment through residential, medical, pharmaceutical, and agricultural sewage [5,6]. Antibiotics in the aquatic environment can cause long-term toxicity to aquatic organisms, impairing their growth, development, and reproductive functions [7]. Effective removal of antibiotic contaminants from water is a hot topic in current environmental research. For high-concentration antibiotic wastewater from pharmaceutical factories, the main treatment methods include advanced oxidation [8], biological treatment [9], photocatalytic degradation [10], and adsorption. In oxidation treatment, the oxidized products may have higher toxicity, causing secondary pollution [11]; biological treatment processes are susceptible to environmental factors and may easily lead to the growth of antibiotic-resistant bacteria [12,13]. Therefore, adsorption is widely used due to its simplicity, low cost, and no generation of other harmful waste [14]. Among various adsorption materials, biochar has broad application prospects due to its regenerability, simple production process, non-toxicity, and abundant raw material resources.

Antibiotic fermentation residues (AFRs) are waste generated during antibiotic production. According to relevant investigations, producing 1 ton of antibiotics generates about 10 tons of antibiotic residues, with an average annual production of 2 × 10^6^ tons [15]. The main components of AFRs include trace antibiotics, metabolites, bacteria of antibiotic-producing bacteria, insoluble components, culture medium, and other nutrients that have not been completely extracted. In addition, the moisture content of AFRs is high, ranging from 79% to 92%, most of which is bound water, with high viscosity, and easy to deteriorate after long-term storage [16]. Antibiotic residues are classified as hazardous waste and can cause water, soil, and air pollution, and harm human health if not disposed of in a timely and effective way [17]. Since 2008, China has listed the antibiotic fermentation residues in the national hazardous waste catalog (HW02-276-001-02) [18]. If improperly treated, AFRs can destroy the ecological environment and endanger human health [19]. For the treatment of AFRs, foreign countries generally use high-temperature incineration [20]. However, due to the large scale of antibiotic production in China and the high cost of incineration, this method cannot be widely implemented. Composting, anaerobic digestion, incineration, and pyrolysis are the commonly used methods in China. The large volume and difficulty of treating AFRs make it a pressing issue to find safe, efficient, and mature treatment technologies to achieve reduction, harmlessness, and recycling of AFRs.

The main objective of this study was to develop a novel porous biochar (OSOB) derived from oxytetracycline fermentation residues (OFRs) and activated by KOH in one step, and its application for the efficient removal of tetracycline. A series of characterization techniques were used to analyze the biochars (OSOBs) and studied the effects of biochar dosage, initial tetracycline concentration, reaction time, and reaction temperature on the adsorption efficiency. Additionally, typical kinetic and isotherm models were used to fit the adsorption process to predict the adsorption mechanism. This study provided a simple, environmentally friendly method to prepare porous biochar from OFRs, with no secondary pollution, aligning with current energy saving and emission reduction policies, transforming waste into valuable resources, and providing an effective solution for recycling antibiotic fermentation residues.

## 2. Materials and Methods

### 2.1. Materials and Chemicals

Oxytetracycline fermentation residues (OFRs) were sourced from Qilian Mountain Pharmaceutical Co., Ltd. (Jiuquan, Gansu Province, China). The residues were dried to a constant weight at 105 °C, ground, and sieved through a 60-mesh screen to obtain fine powder for subsequent use. Tetracycline hydrochloride (TC) was obtained from Shanghai Aladdin Biochemical Technology Co., Ltd. (Shanghai, China). Potassium hydroxide (KOH) was purchased from Komio Chemical Reagent Co., Ltd. (Tianjin, Chnia). Hydrochloric acid was obtained from China National Pharmaceutical Group Corporation. High-purity nitrogen was provided by Kaifeng Wantong Gas Co., Ltd. (Kaifeng, China). All chemical reagents used were of analytical grade.

### 2.2. Preparation of the Activated Biochar

The dried OFRs were mixed with KOH at different mass ratios (1:1, 1:2, 1:3) and ground in an agate mortar until the mixture was uniformly fine and free of large particles. The ground mixture was then placed into a nickel crucible for subsequent pyrolysis. The grinding process should be completed quickly to avoid moisture absorption by KOH. The nickel crucible containing the mixture was placed into a quartz boat and then sequentially inserted into a tubular furnace. Under an N_2_ atmosphere (nitrogen flow rate of 25 mL/min), the temperature was increased at a rate of 2 °C/min to the preset temperature (500 °C, 600 °C, 700 °C) and maintained for 2 h. The samples were then naturally cooled to room temperature in the furnace. The samples were washed several times with 0.1 mol/L HCl until non-alkaline, then washed with deionized water until the pH level reached 7, and finally dried in a vacuum oven at 80 °C for 12 h. The obtained samples were named OSOB-X-Y, where X represents the mass ratio of KOH to OFRs and Y denotes the activation temperature.

### 2.3. The Selection of Different Proportions of Porous Biochar

Preliminary screening of OSOBs was conducted by placing 0.01 g of each porous biochar into a 50 mL centrifuge tube, adding 20 mL of a TC solution (100 mg/L), and placing the tube in a constant temperature shaker at 25 °C and 180 rpm for 24 h. Afterward, the solution was filtered through a 0.45 μm membrane, and the absorbance of the filtrate was measured at 360 nm using an ultraviolet-visible spectrophotometer (752 automatic, Shanghai Jinghua Instrument, Shanghai, China).

The adsorption capacity and removal rate were calculated using Equations (1) and (2), respectively:

(1) $$q_{e}=\frac{\left(C_{e}-C_{0}\right)V}{m}$$

(2) $$R=\frac{\left(C_{e}-C_{0}\right)}{C_{0}}\times 100\%$$

where *C*_0_ (mg/L), *C_e_* (mg/L), *V* (L), and *m* (g) are the initial concentration of TC solution, the equilibrium concentration, the solution volume, and the OSOB-3-700 amount, respectively.

### 2.4. Characterization of Materials

The surface morphology of the OSOB-3-700 was observed using scanning electron microscopy (SEM, Zeiss Merlin Compact, Jena, Germany). The mineral composition and crystal structure of OSOB-3-700 were determined by X-ray Diffraction (XRD, Bruker D8 Advance, Bruker AXS, Saarbruchen, Germany). The specific surface area and pore size distribution of OSOB-3-700 were determined using a specific surface area and pore size analyzer (BET, V-Sorb 2800P, Liaocheng, China). The elemental composition, proportion, and surface characteristics of the OSOB-3-700 were analyzed using X-ray photoelectron spectroscopy (XPS, Thermo Kalpha, Waltham, United States) and elemental analysis (Vario EL cube, Frankfurt, Germany). The weight change of the biochar was measured by the thermogravimetric analyzer (TGA, Mettler TGA/DSC1, Switzerland), which was heated from 30 °C to 900 °C at the heating rate 10 °C/min under an N_2_ atmosphere. The functional groups on the surface of OSOB-3-700 and OSOB-3-700-absorbed TC were analyzed by Fourier-Transform Infrared Spectroscopy (FT-IR, Nexus 470, Ettlingen, Germany).

### 2.5. Batch Adsorption Experiments

To simulate the real wastewater environments, the adsorption capacity of OSOB-3-600 for tetracycline was estimated by examining the effects of the adsorbent dosage (0.125–1.25 g/L), initial tetracycline concentration (250–500 mg/L), contact time (0–1440 min), and experimental temperatures (298 K, 308 K, and 318 K). All batch experiments were conducted in 50 mL centrifuge tubes with a solution volume of 20 mL. The centrifuge tubes were placed in a constant temperature shaker at 180 rpm for 24 h. After adsorption, the solution was filtered through a 0.45 μm membrane, and the absorbance of the filtrate was measured at 360 nm using an ultraviolet-visible spectrophotometer (752 automatic, Shanghai Jinghua Instrument, Shanghai, China). The adsorption capacity and removal rate were calculated using Equations (1) and (2), respectively.

### 2.6. Adsorption Kinetics and Adsorption Isotherms

In a 100 mL tetracycline solution (500 mg/L), 0.025 g of OSOB-3-700 was added to perform adsorption kinetics experiments. After shaking for 5–1440 min, the residual tetracycline concentration was measured. The adsorption capacity of the OSOB-3-700 at a given time (min) was calculated using Equation (3):

(3) $$q_{t}=\frac{\left(C_{t}-C_{0}\right)V}{m}$$

The pseudo-first-order (PFO) and pseudo-second-order (PSO) models were used to study the adsorption kinetics of tetracycline on OSOB-3-600. The non-linear forms of the PFO and PSO models are as follows (Equations (4) and (5)):

(4) $$q_{t}=q_{e}\left(1-e^{-K_{1}t}\right)$$

(5) $$q_{t}=\frac{q_{e}^{2}K_{2}t}{1+K_{2}q_{e}t}$$

where *q_e_*, *q_t_*, *K*_1_ (min^−1^), and *K*_2_ (g/(mg·min)) are the adsorption capacity (mg/g) of the OSOB-3-700 at equilibrium, at a certain time *t* (min), and the adsorption rate constant of PFO and PSO, respectively.

When the adsorption process reached equilibrium, adsorption isotherms could further explore the concentration relationship between the adsorbent and the adsorbate. A mixture of 0.005 g of OSOB-3-700 and 20 mL of TC solution with different TC concentrations (200–500 mg/L) was agitated at 180 rpm for 24 h at temperatures of 25 °C, 35 °C, and 45 °C. The classical isotherm models (Langmuir, Freundlich, and Temkin) were used for analysis, represented by Equations (6)–(8), respectively:

(6) $$q_{e}=\frac{K_{L}q_{m}C_{e}}{1+K_{L}C_{e}}$$

(7) $$q_{e}=K_{F}{C_{e}}^{\frac{1}{n}}$$

(8) $$q_{e}=\frac{RT}{b_{T}}ln(k_{T}C_{e})$$

where *q_e_* (mg/g), *C_e_* (mg/L), *K_L_* (L/mg), *q_m_* (mg/g), *K_F_* (mg/g), *n, b_T_* (J/mol), and *k_T_* (L/mg) represent the adsorption capacity of adsorbent, the concentration of TC solution at equilibrium, the Langmuir constant, the maximum adsorption capacity, the Freundlich sorption coefficient, the adsorption intensity in the adsorption process, the constant related to adsorption heat, and the Temkin isotherm constant, respectively. R (8.314 J/(mol·K)) is the gas constant, and T (K) is the temperature.

### 2.7. The Recyclability Assessment of OCOB-3-700

The recyclability of OSOB-3-700 was investigated through five consecutive adsorption–desorption cycles under the conditions of 0.25 g/L OSOB-3-700 and 250 mg/L initial TC concentration at 180 rpm for 24 h. The used OSOB-3-700 was regenerated using a 0.1 M NaOH solution until no detectable TC concentration remained in the solution. Subsequently, the OSOB-3-700 was rinsed using deionized water until pH = 7 and totally dried at 80 °C.

## 3. Results and Discussion

### 3.1. Analysis of Biochar Selection Results

The OSOBs’ removal rate and the adsorption capacity of TC were measured (shown in Table 1). It can be seen that the porous OSOBs produced at a pyrolysis temperature of 700 °C with a KOH/OFR mass ratio of 3 had a significantly better removal rate and adsorption capacity than other biochars produced at other temperatures and ratios. Therefore, in the subsequent material characterization and adsorption experiments, this temperature and mass ratio of porous biochar (OSOB-3-700) were chosen.

### 3.2. Material Characterization

**Figure 1:**
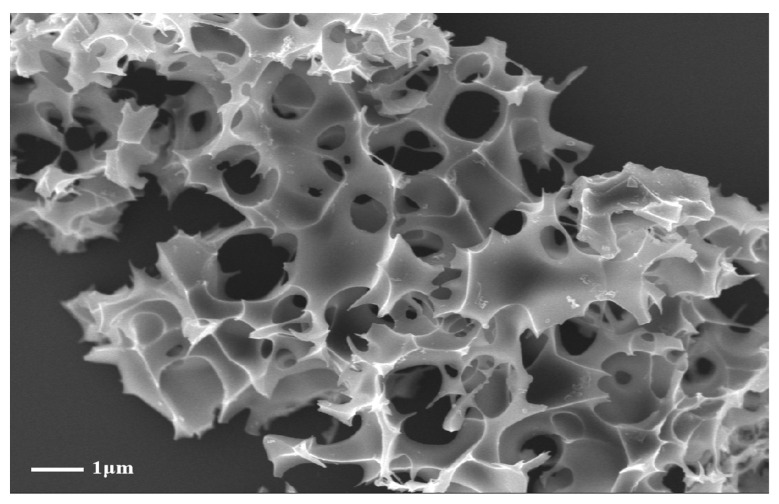
SEM image of OSOB-3-700.

The surface morphology of OSOB-3-700 was observed using a scanning electron microscope (SEM). As shown in Figure 1, after activation with KOH, the surface of OSOB-3-700 particles appeared relatively smooth, with distinct microporous structures. This was due to the reaction between KOH and active oxygen-containing functional groups during pyrolysis, creating numerous vacancies [21]. It produced H_2_ and CO_2_ gases, and these gases escaped during the activation process, forming pores on the surface of material [22]. This change indicated that KOH activation effectively broke the original non-porous framework, forming a porous structure conducive to adsorption. Combining this with the previous biochar selection results, it could be inferred that within a reasonable temperature range, higher temperatures are more favorable for the formation of a porous structure in biochar.

**Figure 2:**
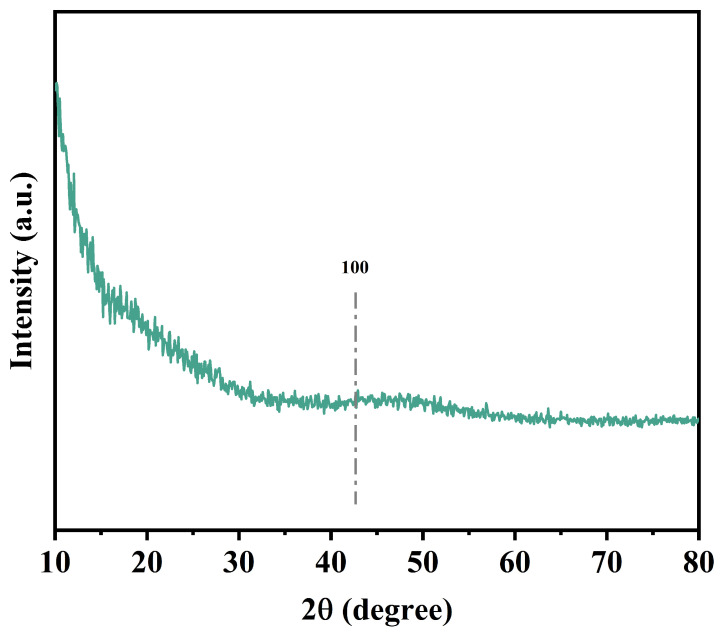
XRD pattern of OSOB-3-700.

The crystal structure and surface morphology could be determined by analyzing the X-ray diffraction pattern of the materials. The XRD pattern of OSOB-3-700 is shown in Figure 2, and there was no obvious diffraction peak. The diffraction results matched the (100) plane diffraction of amorphous graphite carbon, which confirmed that the OSOB-3-700 was amorphous graphite carbon [23]. The broad diffraction peaks suggest that the KOH addition and elevated temperature would enhance the formation of amorphous graphite carbon.

**Figure 3:**
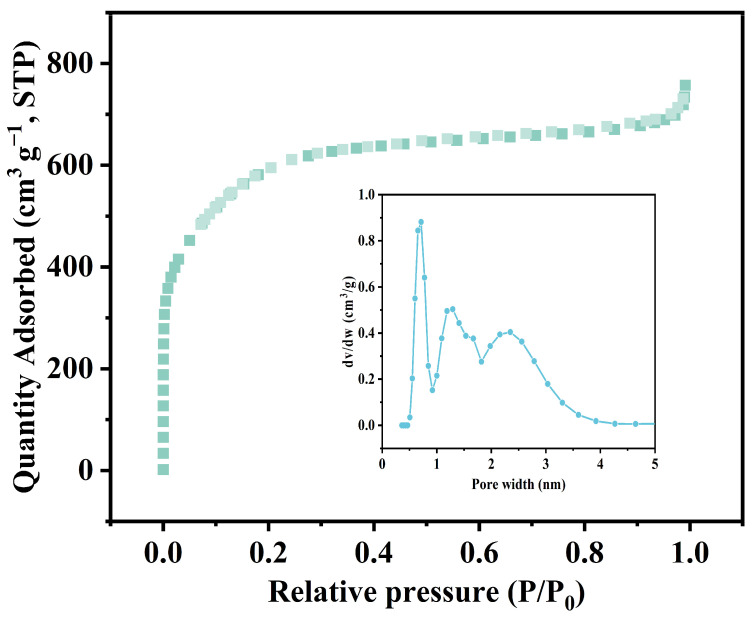
Pore size distribution curve and N_2_ adsorption and desorption isotherms of OSOB-3-700.

BET analysis, Brunauer–Emmett–Teller analysis, is commonly used to analyze the pore size distribution and specific surface area of materials. The adsorption–desorption isotherms and pore size distribution of OSOBs are shown in Table 2 and Figure 3. From Figure 2, it can be seen that the N_2_ adsorption–desorption curve conformed to a Type I isotherm. When P/P_0_ < 0.1, the curve rose rapidly due to micropore filling, indicating significant adsorption of N_2_. When the micropores of OSOB-3-700 reached saturation, the curve showed a plateau turning point, and no significant hysteresis loop was observed, indicating that the pore types were mainly micropores. Similarly, the pore size distribution further confirmed that OSOB-3-700 predominantly consisted of micropores smaller than 1 nm, with numerous and dense pores. The extremely high specific surface area and well-developed pore structure provided an excellent structural foundation for the adsorption of tetracycline by biochar [24].

Table 3 shows the elemental content of the samples. The N content in the OSOB-3-700 sample reached 3.98 wt%, which was significantly higher than the N content in other externally nitrogen-doped biochars (generally 0.35 to 3.42 wt%). This indicated that chemical substances used as nitrogen sources were prone to decomposition at high temperatures, resulting in generally lower N content in the prepared biochar. Therefore, using OFRs as a nitrogen-rich raw material to synthesize nitrogen-doped porous biochar could effectively reduce nitrogen loss. Additionally, the O/C atomic ratio of OSOB-3-700 was less than 0.6, indicating the formation of the carbon matrix and significantly improved aromaticity of OSOB-3-700, which was beneficial for its stable application [25].

**Figure 4:**
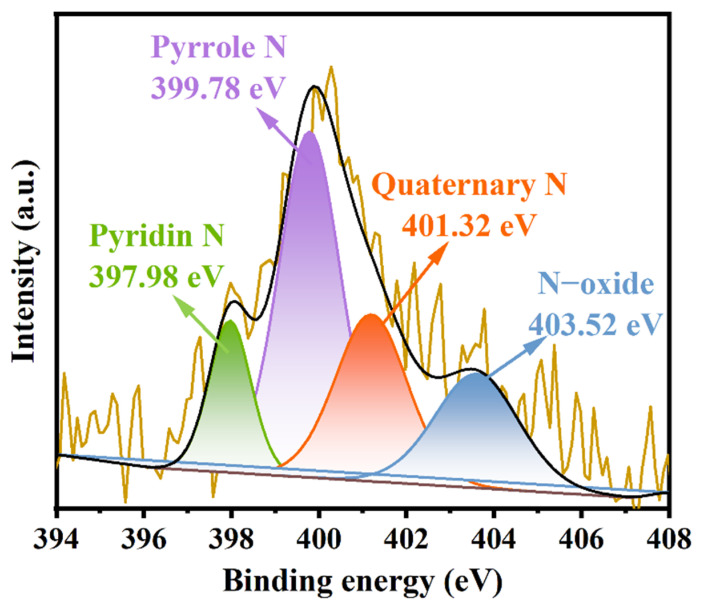
XPS spectrum of OSOB-3-700.

X-ray photoelectron spectroscopy (XPS) analysis can detect very small changes in the chemical composition on the material’s surface [26]. It is an excellent analytical method for studying phase structures, chemical states, and quantitative analysis. As shown in Figure 4, the high-resolution XPS spectrum of OSOB-3-700 could be deconvoluted into four peaks: pyridinic-N (N-6, 397.98 eV), pyrrolic-N (N-5, 399.78 eV), quaternary-N (N-Q, 401.32 eV), and oxidized-N (N-O, 403.52 eV) [27]. Figure 4 indicates that nitrogen-containing groups were mainly present in the form of pyrrolic-N, followed by pyridinic-N, quaternary-N, and oxidized-N. The nitrogen elements in the raw material were successfully incorporated into the OSOB-3-700 through the one-step method. Research showed that N-Q could interact with organic substances through π–π interactions [28]. N-doping also endowed the biochar with a larger specific surface area and rich pore structure, exposing more adsorption active sites and enhancing the adsorption performance of the biochar [29].

**Figure 5:**
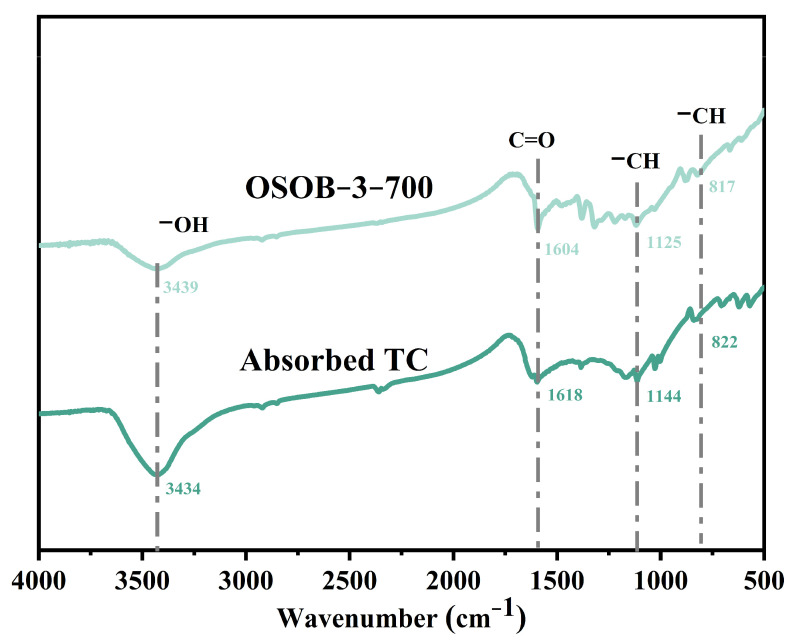
FT-IR spectra of OSOB-3-700 and TC absorbed by OSOB-3-700.

Figure 5 shows the FT-IR of OSOB-3-700 and OSOB-3-700 of TC after adsorption. The peak in the range of 3100~3550 cm^−1^ was an -OH group. The stretching band at 1300–1695 cm^−1^ was mainly attributed to the vibration of C=O, and the peaks at 1151 cm^−1^ and 1306 cm^−1^ were related to C-H [30,31]. Compared with the unused biochar, the absorption peak of OSOB-3-700 at 800–820 cm^−1^ was enhanced after adsorption, which was caused by the C-H vibration on the benzene ring in the TC molecule, indicating that these functional groups were involved in the adsorption process.

**Figure 6:**
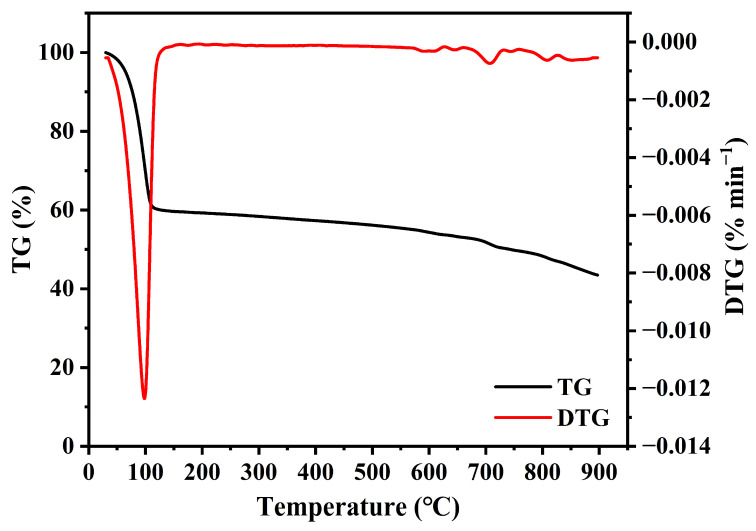
TG and DTG curves of OSOB-3-700.

The thermogravimetric analysis (TGA) was employed to scrutinize the thermal stability of porous biochar materials. Figure 6 indicates that an escalation in pyrolysis temperature precipitated a triphasic mass loss in the samples: initially, the removal of adsorbed water below 200 °C; subsequently, the decomposition of organic components between 200 and 600 °C; and ultimately, the degradation of inorganic constituents above 600 °C [32]. Notably, within the temperature bracket of 30–150 °C, both TG and DTG curves delineated a pronounced weight loss plateau, predominantly attributed to the desorption of moisture from the sample surface. As the temperature surpassed this range, a decrement in the rate of biochar weight reduction was observed, which was indicative of its commendable thermal stability.

### 3.3. Parameters Affecting Adsorption

#### 3.3.1. Effect of Adsorbent Dose

**Figure 7:**
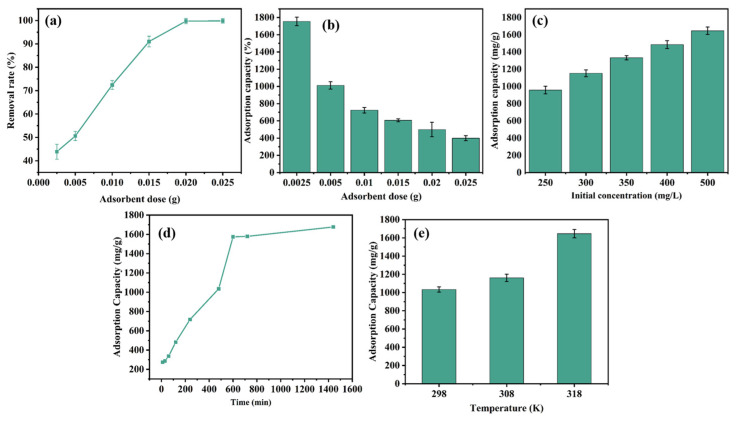
(**a**) Removal rate and (**b**) adsorption capacity on TC with different adsorbent doses (298 K, 24 h, and 500 mg/L); (**c**) Adsorption capacity on TC with different initial concentrations (298 K, 24 h, and 0.25 g/L); (**d**) Effect of contact time on adsorption capacity (298 K, 0.25 g/L, 500 mg/L); (**e**) Effect of temperature on adsorption capacity (24 h, 0.25 g/L, 500 mg/L).

As shown in Figure 7a,b, when the dosage of OSOB-3-700 was 0.0025 g, the removal rate of TC was only 43.86%. When the dosage of OSOB-3-700 increased to 0.025 g, the removal rate of TC reached 99.88%. The removal rate of tetracycline in the solution increased with the increase in the biochar dosage, indicating that as the amount of biochar increased, more adsorption sites were available, leading to more efficient removal of tetracycline from the water [33]. However, as the amount of OSOB-3-700 increased, the adsorption capacity of biochar gradually decreased. This suggested that when the dosage of the adsorbent increased to a certain extent, it could lead to an excess of adsorption sites, resulting in competitive adsorption by OSOB-3-700. Therefore, although the overall removal rate increased, the adsorption capacity decreased [25]. In practical applications, economic efficiency should be considered, and the appropriate amount of biochar should be selected to avoid wasting active adsorption sites. The same dosage was used by Park et al., who used mandarin peel to synthesize biochar for dyes adsorption, and their findings were similar to the present work [34]. Compared with other similar N-doped biochars used for TC adsorption, the OSOB-3-700 synthesized in this work shows a more excellent adsorption capacity for TC. For example, Qu et al., using corn straw, synthesized a nitrogen-doped biochar loaded with FeS (FeS@NBCBM) applied for TC adsorption, and the adsorption capacity for TC was 371.29 mg/g, which was much lower than OSOB-3-700 (1754.57 mg/g) [35].

#### 3.3.2. Effect of Initial Concentration

The initial concentration of tetracycline significantly affected the performance of the adsorbent and could be used as an evaluation indicator of the adsorbent’s suitability in wastewater treatment under varying tetracycline concentrations. The initial concentrations of tetracycline were set at 250, 300, 350, 400, and 500 mg/L to study the effect of initial concentration on adsorption. As shown in Figure 7c, the adsorption capacity of OSOB-3-700 increased with the rise in the initial tetracycline concentration. When the tetracycline concentration increased from 250 mg/L to 500 mg/L, the adsorption capacity increased from 957.77 mg/g to 1645.92 mg/g. As the initial concentration increased, more tetracycline molecules occupied more adsorption sites, leading to a higher adsorption capacity. Mu et al. used tea waste as raw material to prepare biochar through KOH activation pyrolysis and vitamin B6 alkalization. When the initial TC concentration increased from 50 mg/L to 200 mg/L, the removal rate dropped from 100% to below 50% [36]. In contrast, the biochar synthesized in this study only reduced from 100% to 88.52%, indicating that the biochar in this study was more suitable for handling various concentrations of tetracycline, demonstrating more practical application potential.

#### 3.3.3. Effect of Contact Time

As shown in Figure 7d, the entire adsorption process could be divided into a rapid adsorption stage and a slow adsorption stage. The rapid adsorption stage occurred within the first 600 min, followed by the slow adsorption stage. The adsorption of tetracycline by biochar occurred rapidly within 0–600 min, with a removal rate reaching to 78.7%. This was likely because many vacant adsorption sites were available on the surface of OSOB-3-700 at the initial stage, leading to effective adsorption. After 600 min, the increasing trend of the adsorption capacity of OSOB-3-700 slowed down, possibly because the adsorption sites on the biochar surface were nearing saturation, leading to a gradual slowdown in the adsorption rate. As the adsorption time increased, the adsorption sites on the surface of the adsorbent gradually became filled, and the adsorption rate decreased accordingly [37].

#### 3.3.4. Effect of Temperature

The effect of temperature on the adsorption capacity of the adsorbent is crucial for inferring the adsorption mechanism. As shown in Figure 7e, the adsorption capacity of OSOB-3-700 increased with rising reaction temperatures. The elevated reaction temperature accelerated the movement of tetracycline molecules, increasing the collision frequency. This also indicated that the adsorption process is endothermic, requiring more heat to move the adsorbate onto the biochar [38].

### 3.4. Adsorption Kinetics

**Figure 8:**
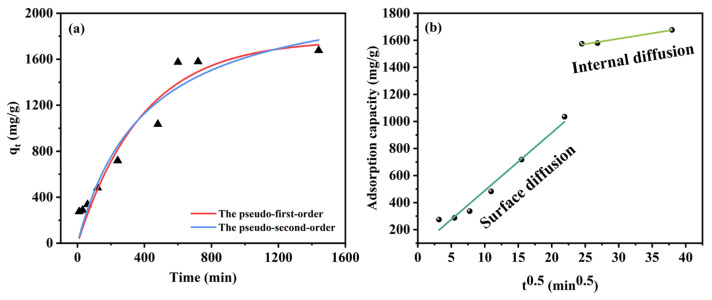
(**a**) Pseudo-first-order and pseudo-second-order fitting curves for TC; (**b**) Intra-particle diffusion kinetic models (289 K, 0.25 g/L, and 500 mg/L).

Adsorption kinetics are essential for studying the adsorption behavior of tetracycline on OSOB-3-700. The obtained data were fitted using two classical kinetic models: pseudo-first-order (PFO) and pseudo-second-order (PSO), shown in Figure 8. The kinetic constants are shown in the Table 4. The adsorption process fitted better with the PFO model (R^2^ = 0.93), indicating that the adsorption of tetracycline on OSOB-3-700 was more consistent with the chemical adsorption mechanism described by the PFO model [39]. In the rapid adsorption phase, tetracycline molecules initially occupied the active sites on the adsorbent surface, forming a monolayer where surface reactions dominated the adsorption process. When the surface adsorption process reached saturation, chemical interactions such as electrostatic adsorption and van der Waals forces became dominant [40]. The maximum adsorption capacity calculated by the PFO model was 2259.02 mg/g, consistent with the actual experimental results. Additionally, the rate constant K being much less than 1 indicated a rapid adsorption process.

The intra-particle diffusion model could further help us understand the adsorption process. The model is represented by Equation (9).

(9) $$q_{t}=K_{i}t^{0.5}+C$$

where *K_i_* (mg/(g·min)) and *C* (mg/g) are the intra-particle diffusion rate constant and the constant about the boundary layer, respectively.

The fitting results are shown in the Table 5. The results indicated that the adsorption process of tetracycline on OSOB-3-700 could be divided into two stages: surface diffusion and intra-particle diffusion. The fact that *K*_1_ > *K*_2_ suggested that the active adsorption sites on the adsorbent were initially occupied by tetracycline molecules, leading to a slower adsorption rate as equilibrium was approached. Additionally, the fitting results indicated that the constant *C* ≠ 0, demonstrating that intra-particle diffusion is not the only rate-limiting step in the adsorption process.

### 3.5. Adsorption Isotherms

**Figure 9:**
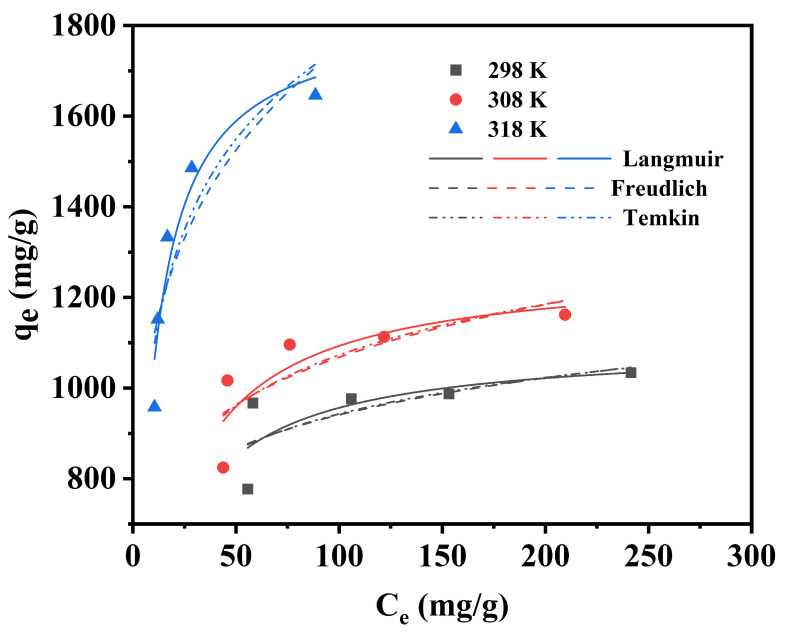
Isotherms fitting curves for TC adsorption (0.25 g/L and 24 h).

Isotherm model fitting is a reliable method for evaluating the feasibility of the adsorption process, evaluating the performance of the adsorbent, and predicting the adsorption mechanism. The classical isothermal models (Langmuir, Freundlich, and Temkin) were used to fit the adsorption process, with the relevant parameters shown in Table 6 and Figure 9. The R^2^ values for the Freundlich model (0.534–0.746) and Temkin model (0.540–0.848) were lower than those for the Langmuir model (0.567–0.930), which can be seen in Table 6 and Figure 6, indicating that the Langmuir model better described the adsorption process of tetracycline on OSOB-3-700 [4]. According to the Langmuir isothermal model, tetracycline underwent monolayer adsorption through chemical interactions at specific homogeneous active sites, which was consistent with the kinetic conclusions [41]. Additionally, the adsorption equilibrium constant K_L_ and its values related to the properties of the adsorbent itself, and the adsorption capacity increased with the increase in K_L_ [42]. At three different temperatures, the maximum adsorption capacities calculated by the Langmuir model were 1096.871, 1270.712, and 1830.624 mg/g, respectively, which were consistent with the actual experimental results, further confirming that the adsorption of tetracycline by the biochar conforms to the Langmuir model.

### 3.6. The Recyclability Assessment of OCOB-3-700

**Figure 10:**
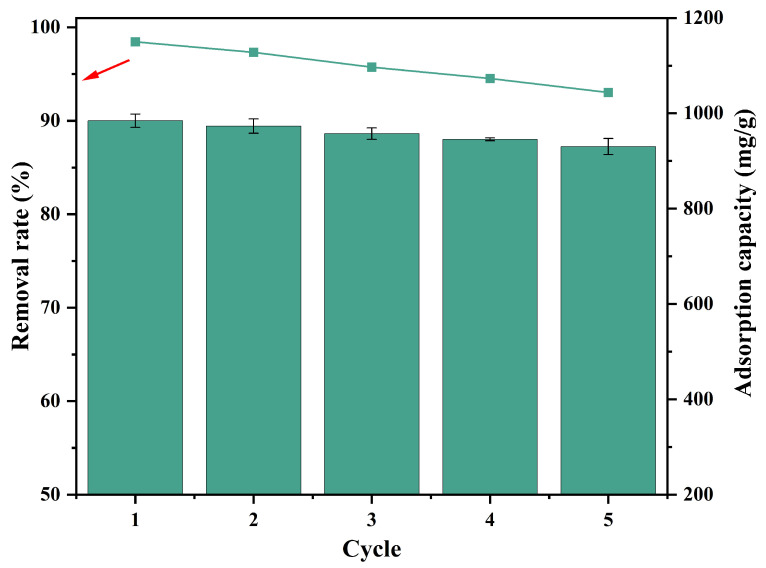
Removal rate and adsorption capacity of TC by OSOB-3-700 in five successive cycles (adsorbent dose = 0.25 g/L, initial concentration = 250 mg/L, at 180 rmp for 24 h).

In practical application, the regeneration performance of biochar is one of the most important factors. Through five consecutive adsorption–desorption experiments, the adsorption capacity of OSOB-3-700 decreased slightly (shown in Figure 10), indicating that it had good regeneration performance. After using NaOH as the eluent for desorption, the adsorption capacity decreased, indicating that the chemisorption was dominant in the adsorption process, and the desorption process destroyed the structure of the biochar surface to some extent [43]. However, the adsorption capacity of biochar treated with HCl as the eluent was only about 50% of the initial, and the adsorption performance was greatly reduced [44]. It was reasonable to use NaOH as the desorption agent, and OSOB-3-700 had good regeneration.

### 3.7. The Comparison between OSOB-3-700 and Other Biochars

In the past decade, many researchers have prepared various new biochars using different biomass raw materials and activators for removing antibiotics from water. By comparing the maximum adsorption capacity calculated by the Langmuir model of OSOB-3-700 with other reported new biochars, as shown in the Table 7, it is evident that OSOB-3-700 has a higher adsorption capacity and is more suitable for treating high-concentration antibiotic wastewater. Furthermore, OSOB-3-700 has the advantage of a relatively simple preparation process without the need for additional acid or metal salt pretreatment. It is worth noting that there is little research on the use of OFRs to prepare porous biochar and apply it to the removal of TC in water.

### 3.8. Possible Adsorption Mechanisms

**Figure 11:**
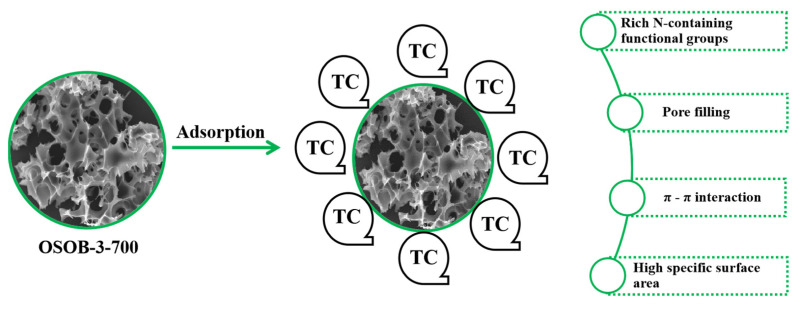
Possible adsorption mechanism of OSOB-3-700 on TC.

To elucidate the adsorption mechanism further, this study conducted an infrared spectroscopic analysis of OSOB-3-700 both before and after the adsorption process, as depicted in Figure 5. A comparative analysis of the FT-IR spectra revealed a pronounced alteration in the intensity of the primary absorption bands, suggesting that the surface functional groups of OSOB-3-700 possess a substantial adsorptive capacity for TC molecules. Kinetic modeling indicated that the adsorption process encompassed both physical and chemical adsorption phenomena. Utilizing SEM and the BET method for surface area analysis, the developed porosity of OSOB-3-700 was confirmed, facilitating the inward diffusion of TC molecules via the pore-filling mechanism [50]. This observation aligned with the fitting outcomes of the intra-particle diffusion model. Furthermore, thermodynamic modeling demonstrated that the adsorption process was endothermic, substantiating the presence of chemical adsorption. A holistic analysis suggested that the adsorption mechanism of TC on OSOB-3-700 likely encompassed both physical effects, such as pore filling, and chemical interactions, including π–π interaction and surface complexation, as illustrated in Figure 11.

## 4. Conclusions

In this study, fresh oxytetracycline fermentation residues were used as raw materials, and KOH was used as a chemical activator to produce biochar with a good adsorption capacity through a one-step pyrolysis method. This material (OSOB-3-700) had characteristics such as high specific surface area, numerous micropores, and abundant surface active sites, making it very suitable for adsorption applications. The adsorption kinetics fitting results indicated that the adsorption of tetracycline on OSOB-3-700 conformed more to the chemical adsorption mechanism described by the PSO model. The experimental results showed that at 298 K, the maximum adsorption capacity of tetracycline on OSOB-3-700 reached 1096.871 mg/g. The adsorption process of tetracycline occurred on the surface of the OSOB-3-700, which was rich in active sites, promoting the adsorption process through electronic exchange and other chemical actions. The adsorption isotherm fitting results showed that the Langmuir model better described the adsorption process of tetracycline on OSOB-3-700. Tetracycline was adsorbed in a monolayer through chemical adsorption at specific homogeneous active sites, consistent with the kinetic conclusions. Therefore, the N-doped porous biochar derived from fresh oxytetracycline fermentation residues was an environmentally friendly material with excellent tetracycline adsorption capacity. This method reduced production costs through waste reuse, had certain economic benefits, and was simple to operate and environmentally friendly, with no secondary pollution. It aligned with current energy saving and emission reduction policies, transforming waste into treasure, and conforms to the concept of sustainable development by enabling the reuse of waste.

## Figures and Tables

**Table 1:** Removal rates and adsorption capacities of OSOBs for tetracycline.

Temperature	KOH/OFR Mass Ratio	Removal Rate	Adsorption Capacity
(°C)		(%)	(mg/g)
500	1	9.60	19.19
500	2	10.81	21.62
500	3	8.26	16.52
600	1	4.19	8.37
600	2	8.99	17.98
600	3	47.62	95.24
700	1	58.65	117.31
700	2	48.08	96.15
700	3	98.87	197.73

**Table 2:** Pore structure parameters of OSOBs.

Samples	S_BET_	V_t_	V_0_	V_t_/V_0_
	(m^2^ g^−1^)	(cm^3^ g^−1^)	(cm^3^ g^−1^)	%
OSOB-1-600	829.27	0.27	0.42	64
OSOB-2-600	1075.64	0.38	0.67	57
OSOB-3-600	1679.61	0.55	0.81	68
OSOB-1-700	1802.60	0.52	1.02	51
OSOB-2-700	1233.55	0.40	0.57	70
OSOB-3-700	2643.78	0.36	1.40	26

**Table 3:** The elemental analysis of samples.

Samples	C	N	O	H	S	O/C	H/C	Yields
	(wt.%)	(wt.%)	(wt.%)	(wt.%)	(wt.%)			%
OSOB-1-500	72.17	5.42	18.92	3.00	0.49	0.26	0.04	20.03
OSOB-2-500	75.62	4.35	16.56	2.89	0.58	0.22	0.04	13.52
OSOB-3-500	69.94	4.67	22.45	2.62	0.32	0.32	0.04	9.61
OSOB-1-600	74.91	6.54	15.68	2.82	0.05	0.21	0.04	21.67
OSOB-2-600	63.36	4.30	29.95	2.39	0.00	0.47	0.04	16.04
OSOB-3-600	68.45	6.56	22.81	2.13	0.05	0.33	0.03	11.48
OSOB-1-700	53.16	3.12	41.79	1.93	0.00	0.79	0.04	29.02
OSOB-2-700	56.29	3.37	38.19	2.15	0.00	0.68	0.04	14.66
OSOB-3-700	59.69	3.98	32.97	1.58	1.78	0.55	0.03	6.91

**Table 4:** Corresponding parameters and coefficients of pseudo-first-order and pseudo-second-order models.

Kinetic Models			
		Pseudo-first-order	Pseudo-second-order
TC	R^2^	0.925	0.931
	q	1769.685	2259.029
	K	0.003	0.000

**Table 5:** Corresponding correlation coefficients of intra-particle diffusion kinetic models.

Sample	K_d1_	C	R^2^	K_d2_	C	R^2^
	(mg/g·min^0.5^)	(mg/g)		(mg/g·min^0.5^)	(mg/g)	
OSOB-3-700	42.731	61.238	0.972	7.995	1372.091	0.986

**Table 6:** Corresponding parameters and coefficients of isotherm models.

	**Langmuir**	**q_m_ (mg/g)**	**K_L_ (L/mg)**	**R^2^**
Temperature (K)	298	1096.871	0.068	0.567
	308	1270.712	0.062	0.724
	318	1830.624	0.132	0.930
	**Freundlich**	**K_F_ (L/mg)**	**1/n**	**R^2^**
Temperature (K)	298	541.789	0.120	0.534
	308	536.814	0.149	0.530
	318	705.342	0.197	0.746
	**Temkin**	**b_T_ (J/mol)**	**k_T_ (L/mg)**	**R^2^**
Temperature (K)	298	21.410	34.556	0.540
	308	15.788	7.459	0.664
	318	9.145	4.250	0.848

**Table 7:** Comparison of various functional biochars for TC removal with OSOB-3-700.

Sample	Original Materials	Method/Activator	Adsorption Capacity (mg/g)	References
OSOB-3-700	Oxytetracycline fermentation residues (OFRs)	Pyrolysis with KOH activation (700 °C, 2 h).	1096.871	This work
Biochar-vitamin B6	Date palm leaves	HClO_3_ impregnation; pyrolysis (500 °C, 1 h); reflux with H_2_O_2_, dry acetonitrile, and vitamin B6 (24 h).	76.92	[45]
SG-ELBC	Eucommia ulmoides (wing husk)	Extracted lignin and mixed with Eucommia ulmoides (70 °C, 2 h); added K_2_SO_3_ (110 °C, 5 h); pyrolyzed (700 °C, 240 min).	944.30	[46]
WS-L	Wheat stalk	Pyrolyzed (600 °C, 5 h); stirred with lignin solution (24 h); calcined (600 °C,5 h).	55.23	[10]
Fe (0.1)-800SBC	Suaeda salsa	Pyrolyzed (800 °C, 2 h); immersed in FeCl_3_ solution, stirred for 3 h, washed into neutral.	70.17	[44]
GPBC-20	Grapefruit peel	The grapefruit peel was mixed with deionized water and then crushed and filtered to separate the filtrate and residue; the residue was pyrolyzed (600 °C, 1 h); soaked in 1:20 g/mL extract (4 h).	34.58	[35]
MSABC	Rice straw	Pyrolysis (300 °C, 1 h); impregnated in NaOH (90 °C, 2 h); treated with glacial acetic acid (10-15 °C, 2 h); added iron salt by co-precipitation method.	98.33	[47]
ZVI@biochar	Hazelnut shell	Mixed with FeCl_3_ solution (1 h); carbonized (849.85 °C, 2 h).	48.30	[48]
AFRB	Penicillin V fermentation residue	Pyrolysis (800 °C, 30 min).	44.05 (for penicillin)	[49]

## Data Availability

Data are contained within the article.
